# Gene expression changes in cerebellum induced by dietary restriction

**DOI:** 10.3389/fnmol.2023.1185665

**Published:** 2023-05-24

**Authors:** Lisanne J. van’t Sant, María B. Birkisdóttir, Rutger A. Ozinga, Ákos Gyenis, Jan H.J. Hoeijmakers, Wilbert P. Vermeij, Dick Jaarsma

**Affiliations:** ^1^Department of Neuroscience, Erasmus MC, Rotterdam, Netherlands; ^2^Princess Máxima Center for Pediatric Oncology, Utrecht, Netherlands; ^3^Oncode Institute, Utrecht, Netherlands; ^4^Cologne Excellence Cluster for Cellular Stress Responses in Aging-Associated Diseases (CECAD), Faculty of Medicine, Institute for Genome Stability in Ageing and Disease, University of Cologne, Cologne, Germany; ^5^Department of Molecular Genetics, Erasmus MC Cancer Institute, Erasmus University Medical Center, Rotterdam, Netherlands

**Keywords:** Purkinje neurons, aging, neurodegeneration, neuroprotective mechanisms, neuronal signaling, DNA repair

## Abstract

**Background:**

Dietary restriction (DR) is a well-established universal anti-aging intervention, and is neuroprotective in multiple models of nervous system disease, including models with cerebellar pathology. The beneficial effects of DR are associated with a rearrangement of gene expression that modulate metabolic and cytoprotective pathways. However, the effect of DR on the cerebellar transcriptome remained to be fully defined.

**Results:**

Here we analyzed the effect of a classical 30% DR protocol on the transcriptome of cerebellar cortex of young-adult male mice using RNAseq. We found that about 5% of expressed genes were differentially expressed in DR cerebellum, the far majority of whom showing subtle expression changes. A large proportion of down-regulated genes are implicated in signaling pathways, in particular pathways associated with neuronal signaling. DR up regulated pathways in large part were associated with cytoprotection and DNA repair. Analysis of the expression of cell-specific gene sets, indicated a strong enrichment of DR down genes in Purkinje cells, while genes specifically associated with granule cells did not show such a preferential down-regulation.

**Conclusion:**

Our data show that DR may have a clear effect on the cerebellar transcriptome inducing a mild shift from physiology towards maintenance and repair, and having cell-type specific effects.

## Introduction

Dietary restriction (DR, also known as caloric restriction) is a well-established anti-aging intervention that consists of reduced food intake without malnutrition (Speakman and Mitchell, 2011; Lee et al., 2021; Acosta-Rodriguez et al., 2022). DR increases health- and lifespan in organisms ranging from yeast to primates with benefits throughout the body (Lee et al., 2021; Green et al., 2022). In rodents and primates, DR has been shown to reduce age-related decline in motor and cognitive function and age-related nervous system pathologies, and to increase resistance to oxidative, metabolic, and excitotoxic insults (Valdez et al., 2010; Wahl et al., 2016; Mattison et al., 2017; van den Boogaard et al., 2021). Furthermore, DR has been shown to reduce neurological deficits and nervous system pathologies in a subset of mouse models for age-related neurodegenerative diseases (Xie et al., 2020; Green et al., 2022; Mitchell and Mitchell, 2022). The anti-aging and neuroprotective effects of DR are thought to be primarily mediated by a network of evolutionary conserved nutrient and energy-sensing pathways that include mTOR, FGF21, insulin/IGF1, AMPK, and sirtuin signaling, and that modulate a variety of intra- and intercellular processes (Mattson and Arumugam, 2018; Komatsu et al., 2019; Lee et al., 2021; Green et al., 2022). Cell-intrinsic neuroprotective mechanisms associated with DR include reduced oxidative stress, improved glucose metabolism, improved proteostasis, increased organelle recycling and enhanced DNA repair (Speakman and Mitchell, 2011; Mattson and Arumugam, 2018; Xie et al., 2020), while cell-extrinsic mechanisms include local factors such as altered glial function and increased neurotrophic signaling, as well as systemic factors such as improved cardiovascular health or immune function (Mattson and Arumugam, 2018; Pluvinage and Wyss-Coray, 2020; Xie et al., 2020; Green et al., 2022). Transcriptome analyses have demonstrated that DR rearranges gene expression in cytoprotective and metabolic pathways, and has differential effects in different tissues and cell types, with relatively limited changes in gene expression in central nervous system as compared to other tissues (Xu et al., 2007; Swindell, 2009; Plank et al., 2012; Barger et al., 2017; Ma et al., 2020). The relatively small effects of DR on gene expression in brain, may reflect the limited bandwidth of brain tissue to adapt its metabolic pathways, because of the continuous high energy demands associated with neuronal function (Harris et al., 2012; Dienel, 2019; Padamsey and Rochefort, 2023).

The cerebellum is affected by a large number of acquired and inherited diseases usually leading to motor and balance abnormalities (Cerminara et al., 2015; Beaudin et al., 2022), and is vulnerably to aging (Andersen et al., 2003). The cerebellum displays different age-related transcriptome and epigenetic changes, and differential vulnerabilities to age-related disorders as compared to forebrain areas (Fraser et al., 2005; Horvath et al., 2015; Liang and Carlson, 2020). Only, a few studies have reported on the effects of DR in the cerebellum. DR has been shown to be beneficial in at least two disease models with cerebellar pathology: In a transgenic mouse model carrying the Atxn3 gene with expanded CAG-repeat region DR reduced cerebellar pathology, potentially mediated by sirtuin signaling (Cunha-Santos et al., 2016). We recently showed that DR strongly delayed Purkinje cell degeneration in progeroid DNA repair-deficient mice (Birkisdottir et al., 2021; Birkisdóttir et al., 2023). The mechanisms underlying the neuroprotective effects of DR in our progeroid mice remain to be defined, although we found that mTOR inhibition seemed not causally involved (Birkisdottir et al., 2021).

To our knowledge there is only a single transcriptome study in the literature that comprehensively examined the effect of DR on gene expression in the cerebellum (Xu et al., 2007). This study suggests that the effect of DR on cerebellar gene expression is minimal, and, in fact, it was concluded that the cerebellar transcriptome was insensitive to DR, yielding no differentially expressed genes (Xu et al., 2007; Swindell, 2009). The study of Xu et al. was performed using microarray’s (Xu et al., 2007), and, hence, the lack of differentially expressed genes in DR cerebellum might be explained by the relative insensitivity of this approach compared to RNAseq (Wang et al., 2014; Zhao et al., 2014). Therefore, to further explore the effect of DR on cerebellum, and to map subtle changes in gene expression, here we analyzed the transcriptome of cerebellar cortex from Ad Libitum (AL) and DR wild-type mice using deep (>50 M reads/sample) sequencing. We found that about 5% of expressed genes were differentially expressed, the far majority of whom showing subtle expression changes in the range of 10%–40% altered expression. The changes in gene expression profiles indicated that DR down-regulated expression of neuronal signaling genes, while upregulated pathways in large part were linked to cytoprotection.

## Methods

### Ethic statements

Animal experiments were performed according to institutional guidelines as overseen by the Animal Welfare Board of the Erasmus MC, following Dutch and EU legislation. Prior to the start of the experiments, a project license for the animal experiments performed for this study was obtained from the Dutch national authority and filed under no. AVD101002015273 (DEC no. 139-12-13, 139-12-18).

### Housing conditions and dietary regimens

Dietary restriction (DR) experiments were performed with wild-type (WT) C57BL6J/FVB F1 hybrid young adult male mice, housed in individual ventilated cages under specific pathogen-free conditions. The environment was controlled with a temperature of 20–22°C and 12 h light:12 h dark cycles. Animals were bred and maintained on AIN93G synthetic pellets (Research Diet Services B.V., Wijk bij Duurstede, Netherlands; gross energy content 4.9 kcal/g dry mass, digestible energy 3.97 kcal/g). Mice were weighed, visually inspected weekly, and scored blindly for gross morphological and motor abnormalities weekly. *Ad libitum*-fed mice (AL, *n* = 6) had unlimited access to food. Animals from the 30% DR group (*n* = 6) received food once a day just before the start of the dark (active) period, Zeitgeber Time (ZT) 12:00. The size of food portions was determined in a prior pilot study where food intake of the AL-fed mice was continuously monitored. Mice on average ate 3.0 g food per day, resulting in 2.1 g/day for 30% DR. Water was freely available to all animals throughout the study. DR was initiated at 7 weeks of age with 10% food reduction, and food was gradually reduced to 30% DR from 9 weeks of age onward as previously published (Vermeij et al., 2016).

### RNA sequencing and analysis

**Figure 1:**
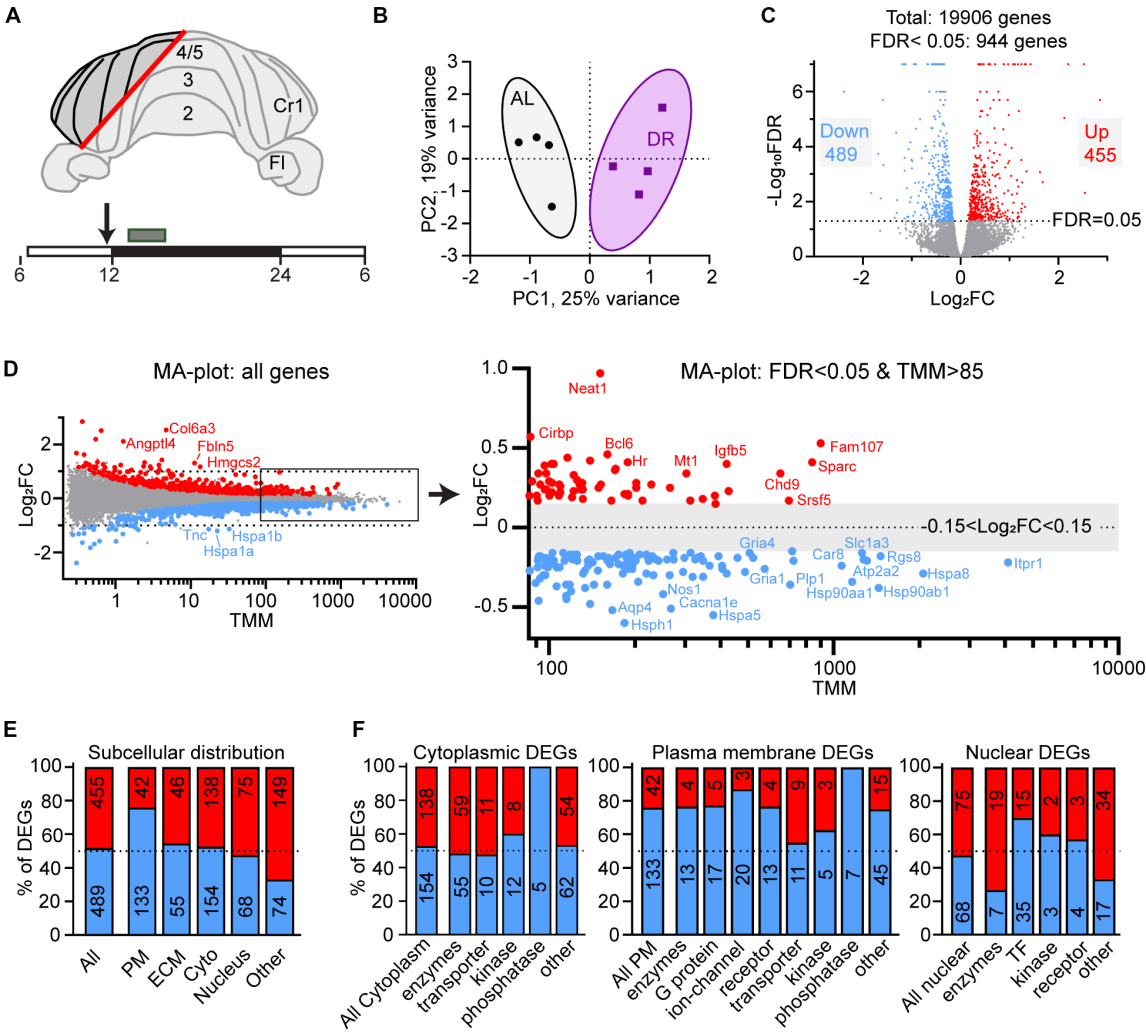
Differential gene expression in DR cerebellum. **(A)** Layout of dissected area of cerebellar cortex from DR and AL wild-type mice used for RNAseq analysis. The box above the time bar indicates the time interval of dissection at the beginning of the dark period (ZT13–ZT16). The arrow indicates the normal time of food delivery, just before the onset of dark period (ZT12) to DR animals. Note that the DR animals did not receive food on the day of dissection. **(B)** Principal component analysis plot showing separation of AL and DR animals. **(C)** Volcano plot showing the distribution of differently expressed genes (DEGs) between AL and DR mice with fold change in expression (log_2_FC) on the x-axis, and false discovery rate (−Log_10_FDR) on the y-axis; DEGs are defined as all genes with FDR < 0.05 (−Log_10_FDR = 1.3, above the dotted line; DR-down genes in blue and DR-up genes in red). **(D)** MA plot illustrating relatively low Log_2_FC values of DEGs of DR versus AL cerebellar cortex. TMM values on the x-axis are means of AL and DR values. The right panel shows a higher magnification of abundantly expressed DEGs (mean TMM ≥ 85) with only DEGs (blue and red dots are down and up in DR DEGs, respectively). Note that the abundantly expressed DR-down DEGs are dominated by heat shock proteins (Hspa5, Hspa8, Hsp90aa1, and Hsp90ab1) and genes linked to glutamatergic signaling (Rgs8, Slc1a3, Gria1, and Gria4) and calcium signaling (Itpr1, Atp2a2, and Car8). **(E,F)** Gene Ontology (GO) annotated subcellular localization **(E)** and functional classes **(F)** of DEGs. PM, plasma membrane; ECM, extra cellular matrix; Cyto, cytolplasm; TF, transcription factors.

At 12 weeks of age, DR and AL animals were sacrificed at the beginning of the dark period (between ZT13 and ZT16), the DR animals without receiving their last meal at ZT12. For RNA isolation a part cerebellar cortex containing multiple folia from the hemisphere, paravermis and vermis, but no cerebellar nuclei (Figure 1A) was rapidly dissected, immediately flash frozen in liquid nitrogen and stored at −80°C.

Total RNA was extracted using QIAzol lysis reagent and TissueLyser LT (Qiagen). For increased purity, miRNeasy Mini Kits (Qiagen) with additional on-column DNase treatment were used. Concentration and quality of RNA was measured by Nanodrop One (Thermo Fisher Scientific, United States) and BioAnalyser 2,100 (Agilent, United States). RNA sequencing was performed with 4 of 6 AL and DR RNA samples selected on the basis of RNA quality (RIN values between 8.9 and 9.3). The TruSeq RNA Library prep kit V2 (Illumina) was used to capture poly(A) RNA from 500 ng total RNA. Subsequently cDNA was made to which single indexed adapters were ligated. To obtain enough material for sequencing a PCR of 13 cycles was performed. Product size was checked on the Labchip GX (Perkin Elmer) and concentrations were measured with picogreen (Invitrogen).

Paired-end sequencing of 2 × 150 bp was performed using the Illumina Hiseq 4,000 platform to obtain at least 15 GB per sample. Removal of sequence adaptors from sequence reads was performed using Trimmomatic (version 0.39). Trimmed reads were aligned to mouse reference genome (annotation: gencode.vM20.annotation.gtf; genome: GRCm38.p6.genome.fa) using STAR (version 2.7.0f). Read counts for each gene were obtained using FeatureCounts (as part of SubRead version 1.6.4). Next, *filterbyExpr* function of EdgeR (version 3.32.1) was used to filter out genes with very low count (average CPM value of 10 or less in both AL and DR groups). This is a common step used to decrease noise in the dataset, as genes with very low counts in deep sequencing are unlikely to be translated into proteins. At the same time, a small difference of counts between groups in lowly expressed genes, can result in large, but often misleading log-fold changes (Chen et al., 2016). Following this step, the genes with sufficiently large counts were TMM-normalized using EdgeR. Normalized genes were used for principal component analysis (PCA), which was performed using the *prcomp* function in R. Lastly, normalized gene counts were quantified, and log2-fold change (logFC) and false discovery rate (FDR) were calculated using EdgeR. All genes with FDR < 0.05 were designated differentially expressed genes (DEGs; Supplementary Table S1). We performed linear correlation analysis between individual sample values of DEGs and selected genes using R and GraphPad Prism software. Heatmaps showing z-scores of gene expression of individual samples were generated in R. All data files have been submitted to the NCBI gene expression omnibus (GEO number: GSE228418).

### Ingenuity pathway analysis (IPA) analysis, subcellular location annotation, and GSEA

Significantly changed pathways associated with differentially expressed genes (DEGs, Supplementary Table S1) were identified using Ingenuity Pathway Analysis (IPA, QIAgen; Supplementary Tables S2). The same program was used to annotate subcellular localizations of the DEGs (Supplementary Table S2).

Gene set enrichment analysis (GSEA) was performed with all expressed genes (19,006 genes, TMM > 0.02) using MSigDB software (GSEA version 3.0; Subramanian et al., 2005), and multiple gene set collections. We first tested gene sets from Hallmark (50 gene sets), KEGG (186 sets) and Reactome (1,654 sets) subsets human curated pathways, and all gene sets from the GO collections (10,532 sets, https://www.gsea-msigdb.org/gsea/msigdb/human/collections.jsp). We only report significant gene sets (*p* < 0.05) whose false discovery rate (FDR) was <0.25 (Supplementary Table S3). Additional gene sets investigated were selected from published datasets with cerebellar cell-specific genes (Rosenberg et al., 2018; Kozareva et al., 2021), neuronal activity-induced genes (Tyssowski et al., 2018), and circadian genes (Zhang et al., 2014). To define cerebellar cortex cell-specific gene sets based on the study of Kozareva et al. (2021), in which each cerebellar cell type is differentiated into multiple subtypes (e.g., 9 subclasses of Purkinje cells), we used different criteria for different cell types as outlined in Supplementary Table S4. For instance, for Purkinje cells we included genes that were found in at least 5 of 9 Purkinje cell subclasses, but were not expressed in other cells.

### Statistical analyses

Statistical analyses were performed using GraphPad Prism Software (San Diego, CA, United States, version 9.5.1). Aggregated *p*-values, where the Fischer method is used to combine statistical values from individual GSEA tests were calculated using the *fisher* function in Rstudio (Version 1.3.1056).

## Results

We applied a 30% DR regimen that we previously have used in progeroid DNA repair-deficient mice starting with 10% restriction at the age of 7 weeks, 20% at 8 weeks, and 30% thereafter (Vermeij et al., 2016). We collected cerebellar tissue at 12 weeks, i.e., 4 weeks after DR onset when the effects of DR on body temperature and metabolic parameters are known to reach stability (Mitchell et al., 2015a). DR animals showed strongly reduced glucose and insulin blood concentrations, increased blood ketones, as well as reduced HDL, LDL and triglyceride blood levels compared to AL animals (Supplementary Figure S1). Body weight was reduced by approximately 30%, while brain weight was relatively preserved, consistent with previous studies (Mitchell et al., 2015b). RNA-seq was performed using an Illumina platform and mapped to the mouse reference genome, yielding 19,906 transcripts. Principal component analysis showed a separation of DR and AL transcriptomes by a single component explaining 25% of the variance (Figure 1B). 5% (944 of 19,006) of expressed genes displayed differential expression (FDR < 0.05), 48% and 52% showing increased (DR-up) versus reduced (DR-down) expression, respectively (Figure 1C, Supplementary Table S1). Large fold changes (log_2_FC > 1) were rare and primarily occurred in DEGs with low mean expression values (TMM < 1; Figure 1D), while the majority of DEGs showed subtle expression changes in the range of 10%–40% (0.15 < Log_2_FC < 0.5, Figure 1D, Supplementary Table S1).

**Figure 2:**
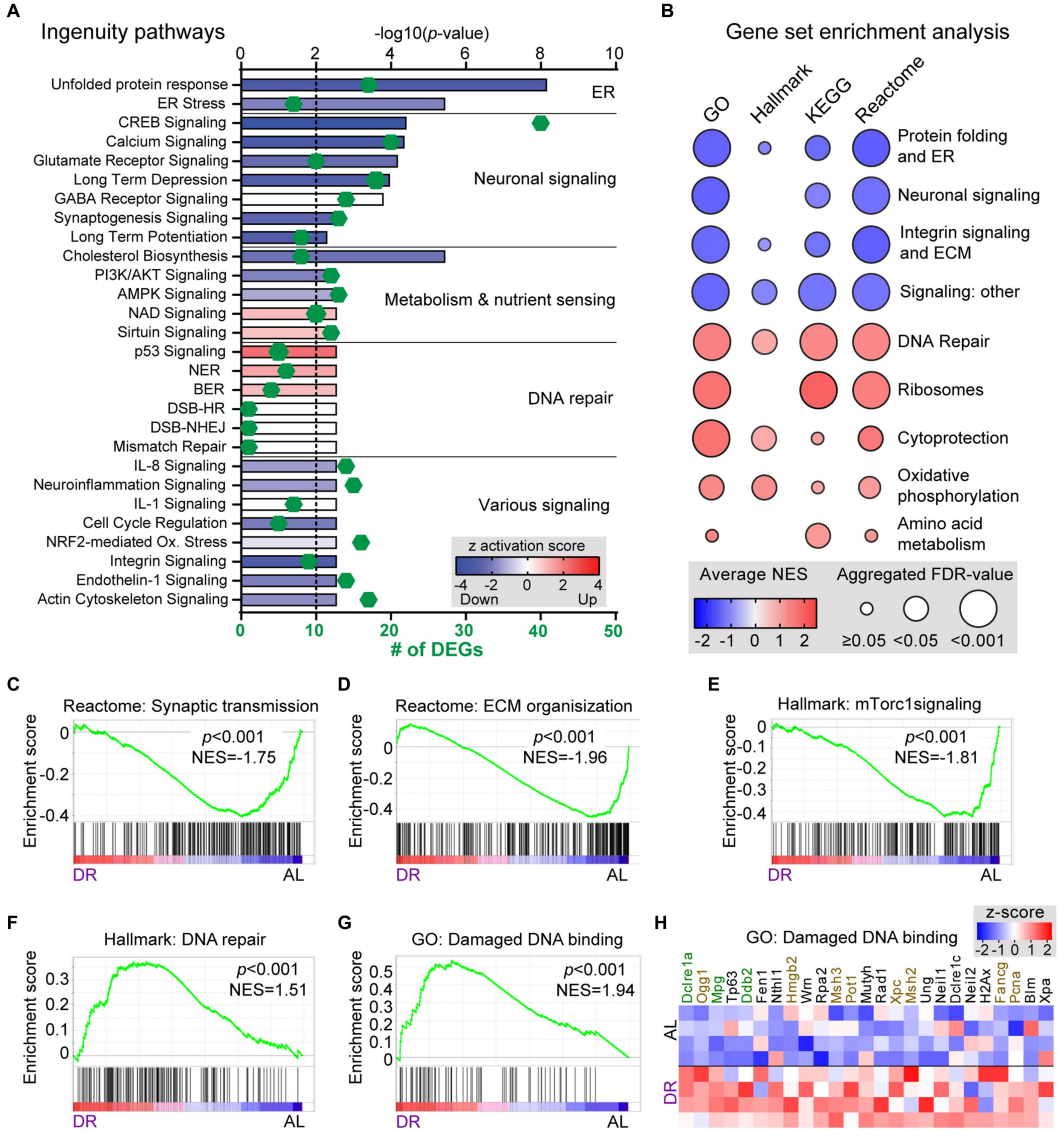
Pathway and GSEA analysis of the effect of DR on cerebellar transcriptome. **(A)** Selection of significantly altered pathways of DEGs as identified with Ingenuity Pathway Analysis. Length of bars indicate the −log_10_(*p*-value) with the dashed line at *p* = 0.01. Color of bars (*z*-score) indicates whether and to which degree pathways are up- (red) or down-regulated (blue). Green diamonds show the number of DEGs associated with the pathway. **(B)** Graph showing summary of results of gene set enrichment analysis (GSEA) with all gene sets from Hallmark, KEGG and Reactome canonical pathways, and Gene Ontology (GO) collections. The graph is based on gene sets with *p* < 0.05 and FDR < 0.25 categorized as outlined in Supplementary Table S3. **(C–E)** Exemplary GSEA graphs illustrating enrichment of down-regulated genes in gene sets associated with synaptic transmission **(C)**, extra cellular matrix **(D)**, and mTorc1 signaling **(E)** in DR cerebellar cortex. **(F–H)** GSEA graphs **(F,G)** and heat map [**(H)** with *z*-scores of genes of **(G)**] illustrating enrichment of up-regulated genes in gene sets associated with DNA repair pathways in DR cerebellum. In **(H)** genes in green and brown have FDR and *p* values <0.05, respectively.

Gene ontology (GO) assessment of the DEGs revealed specific changes according to subcellular distribution with an increased relative abundance of DR-down DEGs in plasma membrane genes (76% down; Figure 1E), including ion channels, receptors and G-proteins (Figure 1F). Accordingly, Ingenuity pathway analysis (IPA) of DEGs identified neuronal signaling pathways, including calcium signaling and glutamate receptor signaling, and synaptic plasticity, among the top enriched pathways of down regulated genes by DR (Figure 2A; Supplementary Table S2). Down-regulated processes, further consisted of additional signaling pathways, including integrin signaling, nutrient-sensing, and inflammatory signaling, as well as protein folding and ER stress (Figure 2A; Supplementary Table S2). Upregulated pathways included NAD signaling and DNA repair pathways (Figure 2A; Supplementary Table S2).

To further define down- and upregulated processes in DR cerebellum we compared DR vs. AL gene expression of all genes using gene set enrichment analysis (GSEA; Subramanian et al., 2005) with all gene sets from Hallmark, KEGG and Reactome curated pathways, and GO collections (Figure 2B; Supplementary Table S3). Consistent with IPA pathway analysis of DEGs, gene sets showing overall reduced expression in DR were predominantly associated with nutrient-sensing, inflammatory, extracellular matrix, and neuronal signaling (Figures 2B–E and Supplementary Figures S2A–D; Supplementary Table S3). Since the global down-regulation of neuronal signaling pathways may be associated with reduced neuronal activity, we also performed GSEA with gene sets activated by neuronal activity (Tyssowski et al., 2018). This analysis showed reduced expression of neuronal-activity regulated genes in DR cerebellum, consistent with attenuated neuronal activity (Supplementary Figure S2F). Gene sets with increased expression in DR predominantly consisted of ribosomal genes, DNA repair genes, and gene sets associated with cytoprotective pathways (Figures 2B,F–H, Supplementary Figure S2G,H; Supplementary Table S3). Top enriched DNA repair gene sets included base excision repair, interstrand crosslink repair and mismatch repair pathways, as well as processes like DNA damage recognition and binding (Figures 2F–H, Supplementary Figure S2G; Supplementary Table S3). Finally, GSEA analysis indicated that gene sets of metabolic pathways were relatively unaffected by DR, except for mild upregulation of some gene sets associated with oxidative phosphorylation and amino acid metabolism (Figure 2B; Supplementary Figure S2; Supplementary Table S3).

**Figure 3:**
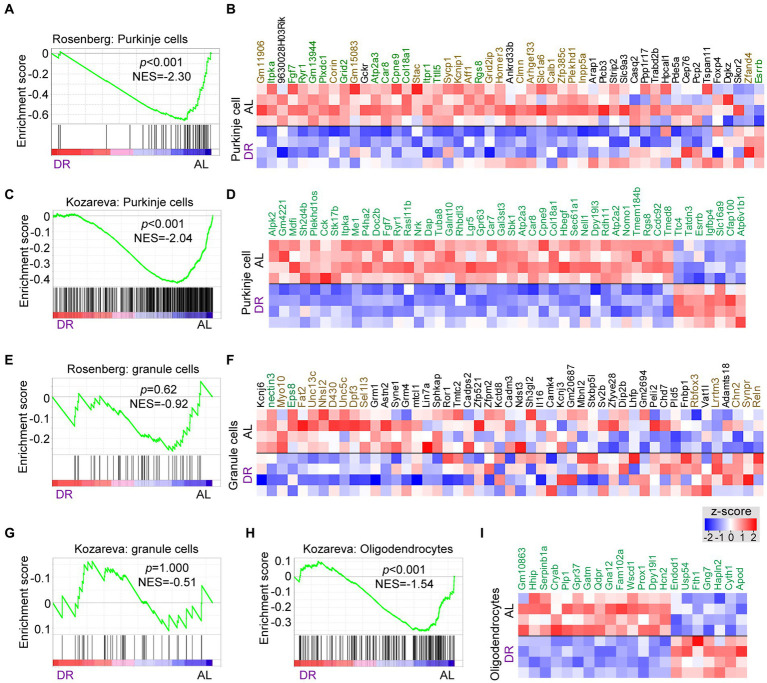
Cell specific effects of DR in cerebellum. GSEA graphs and heat maps gene sets specifically expressed in Purkinje cells **(A–D)**, granule cells **(E–G)** and oligodendrocytes **(H–I)**, based on gene lists from Rosenberg et al. (2018) and Kozareva et al. (2021) (see Supplementary Table S4 for gene sets). Heat maps in **(D)** and **(I)** only show differentially expressed genes (DEGs, in green), while the heat maps in **(B)** and **(F)** shows all genes of the Rosenberg Purkinje and granule cell lists, with genes in green and brown showing FDR < 0.05 and *p* < 0.05, respectively, in DR versus AL comparison. Note, enrichment of down-regulated genes (*p* < 0.01) in gene sets associated with Purkinje cells **(A,C)** and oligodendrocytes **(H)**, while no specific enrichments occur in granule cell gene sets.

Many DR-down DEGs associated with neuronal signaling, including Abdh2, Atp2a3, Baiap2, Car8, Grid2, Itpr1, Prkg1, Ryr1, Shank2, Shisha6, and Trpc3, are preferentially expressed in Purkinje cells. Further analysis of changes in Purkinje cells using GSEA analysis with Purkinje cell-specific gene lists (Rosenberg et al., 2018; Kozareva et al., 2021) indicated a preferential down-regulation of Purkinje cell genes by DR (Figures 3A–D; Supplementary Table S4). Instead, no enrichment of up or down genes were observed in granule cells, representing another major class of neurons in the cerebellar cortex (Figures 3E–G), while variable changes were observed with gene sets associated with glia cells (Figures 3H,I; Supplementary Figure S4). Thus GSEA analysis with cell-specific gene sets indicates that DR differentially impacts on Purkinje cells as compared to granule cells causing a preferential down-regulation of Purkinje cell-specific genes. Analysis of cell-specific gene sets further enabled to identify cell-specific pathways in glia cells altered by DR, including altered myelin regulation (Hhip, Plp1, Gpr37, Prox1, Hcn2, and Fth1) in oligodendrocytes (Figures 3H,I), and altered Shh signaling (Gli1, Ptch1, Ptch2, Trib2, and Bcl6) in Bergman glia cells (Supplementary Figure S4).

**Figure 4:**
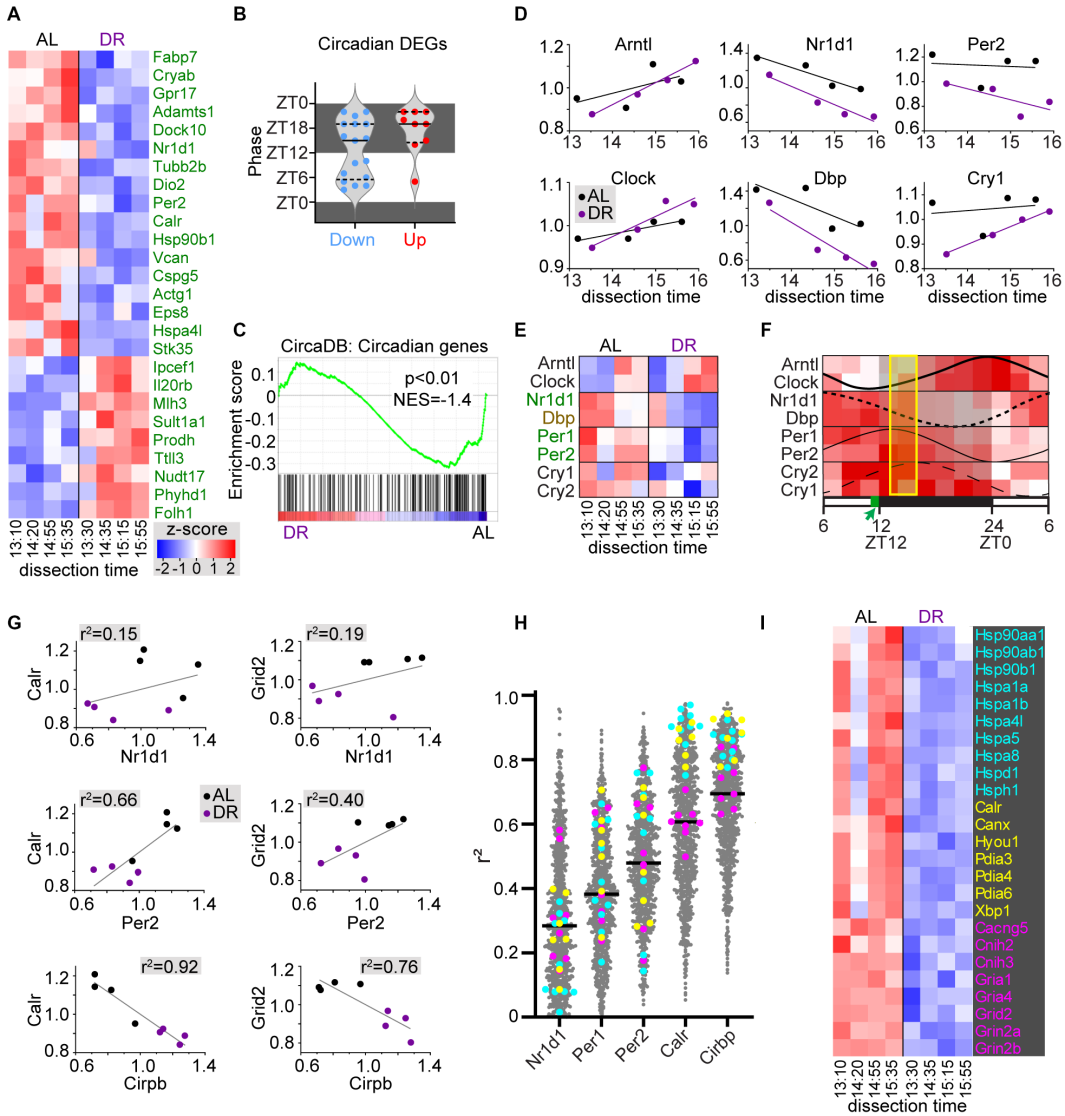
DR induces changes in circadian cerebellar genes and heat shock chaperones. **(A,B)** Heatmap **(A)** and bar graph with peak phases **(B)** of differentially expressed circadian genes in DR versus AL cerebellar cortex. Dissection time [x-axis in **(A)**] is indicated as zeitgeber time (ZT). **(C)** GSEA plot of cerebellar circadian genes (i.e., genes from CircaDB mouse cerebellar database with JTK *q*-value < 0.05) in DR versus AL cerebellar cortex. **(D–F)** x-y plots **(D)** and heat map **(E)** of core clock genes expression related to time of dissection show that changes in core clock gene expression throughout the dissection period in both AL and DR cerebellum are consistent with oscillation phases of core clock gene expression in cerebellum as illustrated in panel **(F)** that is based on Supplementary Figure S5 of Zhang et al. (2014). Genes in green and brown have FDR < 0.05 and *p* < 0.05, respectively in DR versus AL comparison. The yellow box in **(F)** indicate the period of dissection (ZT13–ZT16), and the green box in the time bar indicates the time of daily food delivery to DR animals. **(G)** x-y plots with linear correlation lines **(G)** illustrate a poor correlation between sample values of 2 exemplary DEGs (Calr and Grid2) and differentially expressed core clock genes Nr1d1 and Per2. Instead, Calr values show a strong negative correlation with the hypothermia-induced factor Cirbp that shows increased expression in DR cerebellum. Further linear correlation analysis of all DEGs [grey dots in **(H)**] versus differentially expressed core clock genes and Cirbp, indicates stronger correlations with Cirbp compared than with Nr1d1, Per1, and Per2, in particular for all differentially expressed heat shock proteins [cyan in **(H,I)**], and ER chaperones [yellow in **(H,I)**]. Genes in pink in **(H,I)** are glutamate receptor subunits and adaptors.

Since DR may affect circadian physiology and metabolism, and have tissue-specific effects on circadian gene expression (Speakman and Mitchell, 2011; Greco and Sassone-Corsi, 2019; Acosta-Rodriguez et al., 2022), we looked at the effect of DR on a list of 220 cerebellar circadian genes from the CircaDB database (http://circadb.hogeneschlab.org/; Zhang et al., 2014). A subset of these (26 of 220) were DEGs, most of which (17 of 26) were DR-down DEGs with peak phases both in the dark and the light period (Figures 4A,B). Instead, the DR-up circadian DEGs (9 of 26) predominantly have a peak phase in the dark (Figure 4B). Also, GSEA analysis indicated a trend of reduced expression of circadian genes in DR cerebellum (Figure 4C). DR down DEGs included negative feedback loop factors (Nr1d1, Per1, and Per2) of the core clock genes, while the key positive regulators Arntl (Bmal1) and Clock showed no differential expression in DR cerebellum. Interestingly, plotting of core clock gene expression values of individual samples against dissection time, revealed gene-dependent upward (Arntl, Clock, and Cry1) or downward (Nr1d1, Dbp, and Per2) changes in expression throughout the dissection time window (Figures 4D,E). These changes in time were consistent with expected directionality based on documented oscillation phases of cerebellar core clock genes (Figure 4F; Zhang et al., 2014), and support the notion that our DR procedure does not have a major effect on the phase of core clock genes as previously documented (Mendoza et al., 2010; Acosta-Rodriguez et al., 2022), and unlike other feeding schemes such as food delivery halfway the light period (Mendoza et al., 2010; Acosta-Rodriguez et al., 2022). Importantly, the analysis of core clock gene expression of individual samples also indicates that the variability between samples from the same treatment groups embodies biological meaningful information. Linear correlation analysis of individual sample values suggested relatively poor correlations between expression of differentially expressed core clock genes (Nr1d, Per1, Per2) and other DEGs (Figures 4G,H). Instead, expression of many DEGs showed a better linear correlation with Cirbp mRNA, a hypothermia-induced factor that showed increased expression in DR cerebellum (Figures 4G,H). For instance, DR-down heat shock proteins and ER chaperones showed a strong negative correlation with Cirbp mRNA levels (Figures 4G–I), indicating that these changes in molecular chaperone expression may reflect changes in body temperature triggered by DR (Mitchell et al., 2015a; Guijas et al., 2020), and further illustrating that the variability between samples in our RNAseq dataset may embody biological meaningful information.

## Discussion

In this study we show that a classical 30% DR protocol with strong anti-aging effects and neuroprotection in multiple neurodegenerative models, including models with cerebellar pathology (Speakman and Mitchell, 2011; Cunha-Santos et al., 2016; Vermeij et al., 2016; Xie et al., 2020; Acosta-Rodriguez et al., 2022; Birkisdóttir et al., 2023), does have a distinct and important effect on gene expression in cerebellar cortex, with about 5% of the expressed genes showing differential expression compared to AL-fed control mice. The far majority of DEGs showed relatively small expression changes in the range of 10–40% increased or reduced expression, which may explain why no consistent gene expression changes have been observed in DR cerebellum in previous microarray experiments (Xu et al., 2007; Swindell, 2009) with reduced sensitivity compared to the deep sequencing approach of our study. The overall picture that emerges from the DR-induced gene expression changes is a mild shift from physiology towards maintenance and repair (Finkel, 2015; Vermeij et al., 2016). Thus, a considerable proportion of down-regulated genes are involved in neuronal signaling, while many upregulated genes are linked to cytoprotective mechanisms, including DNA repair. Upregulation of cytoprotective and DNA repair pathways is consistent with data from other brain areas and tissues (Xu et al., 2007; Swindell, 2009; Plank et al., 2012; Barger et al., 2017; Ma et al., 2020; Wahl and LaRocca, 2021), and can be mechanistically linked to the neuroprotective effect of DR in cerebellar neurodegeneration mouse models (Cunha-Santos et al., 2016; Birkisdottir et al., 2021; Birkisdóttir et al., 2023). Upregulation of DNA repair pathways, for instance, may reduce the accumulation DNA damage in aging (Heydari et al., 2007; Van Houten et al., 2018), and contribute to the strong beneficial effect of DR in DNA-repair-deficient accelerated aging mouse models (Vermeij et al., 2016; Birkisdottir et al., 2021; Birkisdóttir et al., 2023).

The nervous system requires continuous supply of glucose and oxygen, and has limited opportunities to save energy, which results in reallocation of energy from other tissues to the brain in condition of scarcity (Harris et al., 2012; Dienel, 2019; Padamsey and Rochefort, 2023). Brain energy consumption may be reducing by reducing body temperature and physical activity, by modulating sleep, and, in mice, by inducing torpor, a state in which whole-body metabolism and neuronal activity is substantially reduced (Speakman and Mitchell, 2011; Sonntag and Arendt, 2019; Padamsey and Rochefort, 2023). Interestingly, recently it has been found that circuitries in neocortex in conditions of food restriction can adapt their properties, and reduce their energy demand via a leptin-dependent mechanism and involving adaptation of AMPA-receptor signaling (Padamsey et al., 2022). The reduced expression in synaptic and neuron signaling genes as we observed in DR cerebellum may reflect a similar adaptation to reduce energy consumption. Interestingly, reduced synaptic gene expression predicts a longer lifespan among healthy aging individuals (Zullo et al., 2019), raising the possibility that adapting neuronal excitation and synaptic function may be one of the mechanisms by which DR exerts its anti-aging and neuroprotective effects (Aron et al., 2022). Our data indicate that a large proportion of down-regulated neuronal signaling genes is expressed by Purkinje cells, representing large continuously firing neurons. Interestingly, Purkinje cells can be grouped in different subtypes with distinct firing properties coupled to differences in metabolic and signaling gene expression, and differences in disease vulnerabilities (Cerminara et al., 2015). The precise impact, of DR on firing properties and gene expression of these Purkinje cell subtypes remains to be determined in future studies.

Our demonstration that DR indeed significantly alters the cerebellar transcriptome, provides a starting point for further analyses of cerebellar changes triggered by DR. In this study we examined young adult male C57BL6J/FVB F1 hybrid mice at a single time window of the diurnal cycle. Thus, our findings remain to be examined in cohorts with female mice, with mice with different genetic background, and with old mice (Acosta-Rodriguez et al., 2022; Mitchell and Mitchell, 2022). Furthermore, analysis of gene expression at multiple diurnal time points, may provide a dynamic picture of DR induced transcriptome changes throughout the day, and could expose how transcriptome changes relate to DR-induced diurnal changes in metabolism, body temperature, as well as sleeping and physical activity patterns (Nelson and Halberg, 1986; Speakman and Mitchell, 2011; Greco and Sassone-Corsi, 2019; Guijas et al., 2020; Acosta-Rodriguez et al., 2022). Finally, cell specific approaches, for instance *via* cell specific isolation of ribosomes or nuclei (Doyle et al., 2008; Chen et al., 2022) may further expose cell specific pathways induced by DR in the cerebellum.

## Data availability statement

The original contributions presented in the study are publicly available. This data can be found here: https://www.ncbi.nlm.nih.gov/, GSE228418.

## Ethics statement

The animal study was reviewed and approved by Animal Welfare Board of the Erasmus MC. Written informed consent was obtained from the owners for the participation of their animals in this study.

## Author contributions

WV and DJ conceptualized and designed the experiments. MB, LvS, ÁG, RO, WV, and DJ performed transcriptomics analysis. MB, LvS, WV, and DJ wrote the manuscript. JH contributed to editing the manuscript. All authors contributed to the article and approved the submitted version.

## Funding

We acknowledge financial support of the National Institute of Health (NIH)/National Institute of Aging (NIA) (AG17242), European Research Council Advanced Grants Dam2Age (to JH), ONCODE supported by the Dutch Cancer Society, ADPS Longevity Research Award (to WV), Memorabel (ZonMW 733050810), BBoL (NWO-ENW 737.016.015), Deutsche Forschungsgemeinschaft (DFG, German Research Foundation—Project-ID 73111208—SFB 829), Regiodeal Foodvalley (162135) and the European Joint Programme Rare Diseases (TC-NER RD20-113).

## Conflict of interest

The authors declare that the research was conducted in the absence of any commercial or financial relationships that could be construed as a potential conflict of interest.

## Publisher’s note

All claims expressed in this article are solely those of the authors and do not necessarily represent those of their affiliated organizations, or those of the publisher, the editors and the reviewers. Any product that may be evaluated in this article, or claim that may be made by its manufacturer, is not guaranteed or endorsed by the publisher.
