# Supplementary material for: Gene expression changes in cerebellum induced by dietary restriction
Source: Front Mol Neurosci. 2023 May 24;16:1185665. doi: 10.3389/fnmol.2023.1185665 (PMC10244750; doi:10.3389/fnmol.2023.1185665)
Supplement: Supplementary file 5 [file Data_Sheet_1.pdf]

<u>**Supplementary Figures**</u>

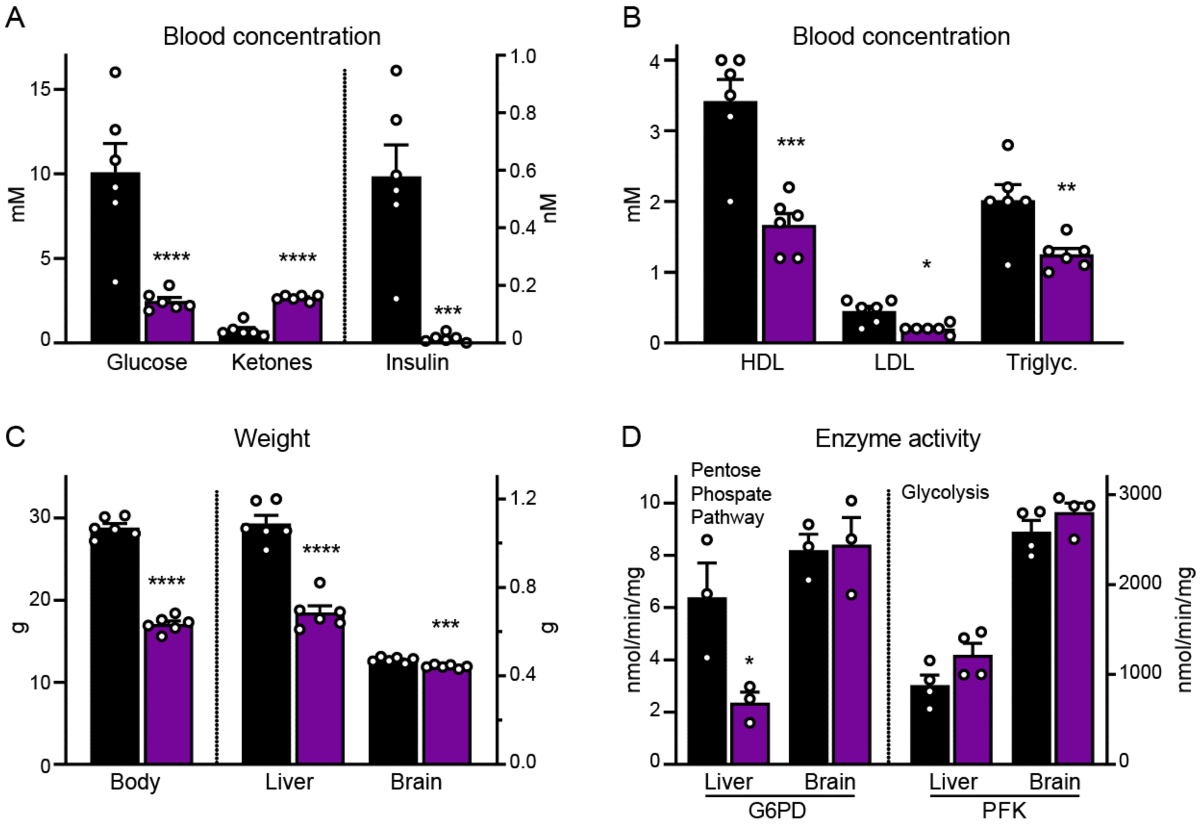

Supplementary Figure S1. Altered weight and metabolic parameters in DR vs AL mice

A,B. Reduced glucose, insulin (A), LDL and HDL cholesterol, and triglycerides (B) levels in blood of DR animals, while the concentration of ketone bodies (A) is increased.

C. Substantial loss of body weight and liver weight, while brain weight is relatively preserved.

D. Reduced glucose-6-phosphate dehydrogenase (G6PD) activity in liver, but not brain, homogenate from DR animals, while no change in phosphofructokinase activity was observed.

****, ***, **, * p<0.0001, <0.001, <0.01, <0.05 unpaired students *t*-test.

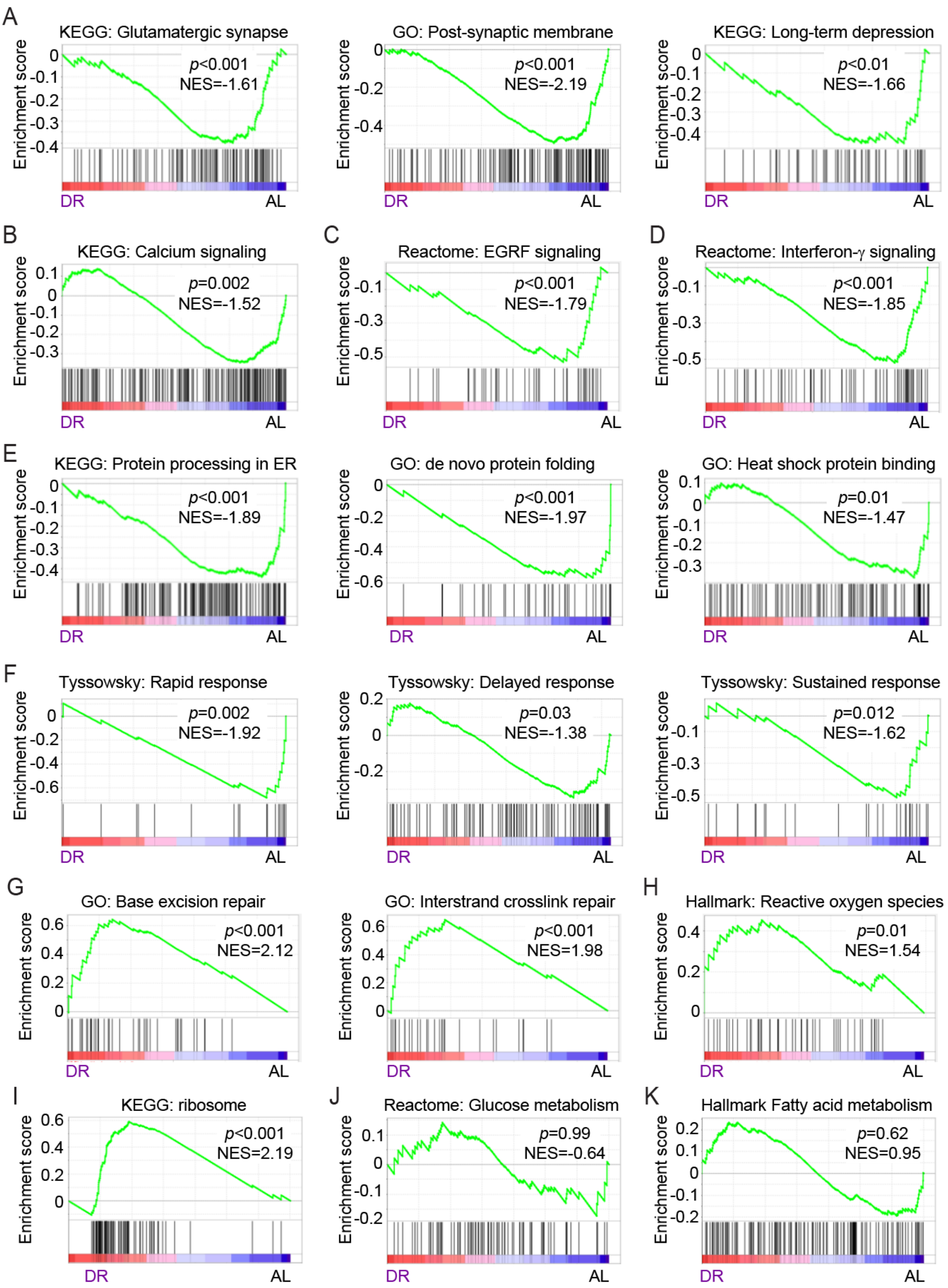

Supplementary Figure S2. Exemplary GSEA graphs illustrating down- and up-regulated gene sets in DR vs AL cerebellum

Neuronal activity-induced gene sets (F) are from Tyssowski et al. (Tyssowski et al., 2018), while other gene sets are from GO, KEGG, Reactome and Hallmark collections.

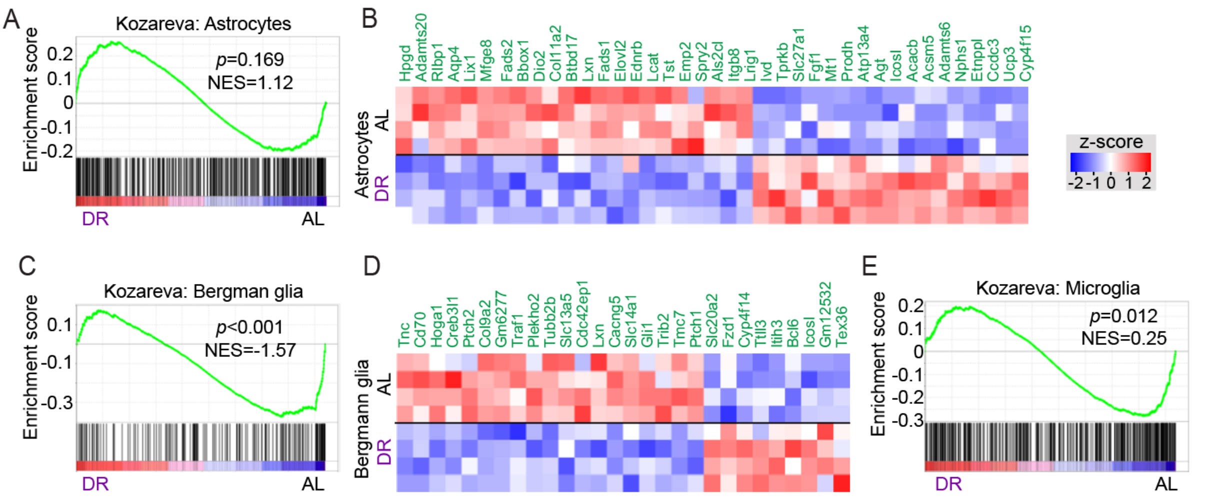

Supplementary Figure S3. GSEA analysis of glia-specific gene sets.

GSEA graphs and heat maps of cell-specific genes based on gene lists of Kozareva et al. (2021) (see table S4 for gene sets). Heat maps (B, D) show z-scores of differentially expressed genes of gene sets of A and C, respectively. Note, enrichment of down-regulated genes (*p*<0.001) in gene sets associated with Bergman glia (C, D).
